# Supplementary material for: mitoTev‐TALE: a monomeric DNA editing enzyme to reduce mutant mitochondrial DNA levels
Source: EMBO Mol Med. 2018 Jul 16;10(9):e8084. doi: 10.15252/emmm.201708084 (PMC6127889; doi:10.15252/emmm.201708084)
Supplement: Supplementary file 1 — Appendix [file EMMM-10-e8084-s001.pdf]

APPENDIX

APPENDIX TABLE S1
DETAILED STATISTICAL RESULTS

FIGURE 2B
FIGURE 2D
FIGURE 2E
FIGURE 2F

FIGURE 3B
FIGURE 3C
FIGURE 3F
FIGURE 3G

FIGURE 4B
FIGURE 4C
FIGURE 4D
FIGURE 4E

EXPANDED FIGURE EV1D

Figure 2B				
		N value	exact *p* value	asterics
	UNT	11	N/A	
	Black	5	0.4988	ns
	Yellow	5	6.11362E-06	****
	GFP-+	12	0.027629843	*
	GFP++	12	3.12849E-06	****

Figure 2D				
		N value	exact *p* value	asterics
1 day	UNT	3	N/A	
	GFP-+	3	0.0145	*
	GFP++	3	0.0016	**
15 days	UNT	3	N/A	
	GFP-+	3	0.2317	ns
	GFP++	3	0.006	**

Figure 2E				
		N value	exact *p* value	asterics
1 day	UNT	6	N/A	
	GFP-+	5	0.852	ns
	GFP++	6	0.0013	**
15 days	UNT	4	N/A	
	GFP-+	3	0.5998	ns
	GFP++	3	0.0242	*

Figure 2F				
		N value	exact *p* value	asterics
1 day	UNT	6	N/A	
	GFP-+	6	0.1692	ns
	GFP++	6	0.3796	ns

Figure 3B				
		N value	exact *p* value	asterics
2-3 days	UNT	8	N/A	
	GFP-+	8	0.2533	ns
	GFP++	8	0.0002	***
15-20 days	UNT	7	N/A	
	GFP-+	7	0.0227	*
	GFP++	7	1.99367E-05	****
23-27 days	UNT	6	N/A	
	GFP-+	6	0.0269	*
	GFP++	6	0.0002	***

Figure 3C				
		N value	exact *p* value	asterics
2-3 days	UNT	7	N/A	
	GFP-+	7	0.131	ns
	GFP++	7	8.96389E-05	****
15-20 days	UNT	5	N/A	
	GFP-+	6	0.9012	ns
	GFP++	6	0.00051	***
23-27 days	UNT	6	N/A	
	GFP-+	6	0.382	ns
	GFP++	6	0.00251	**

Figure 3F				
		N value	exact *p* value	asterics
2 day	UNT	4	N/A	
	GFP-+	4	0.0036	**
	GFP++	4	0.0145	*
	GFP+++	4	0.0001	***
15 days	UNT	3	N/A	
	GFP-+	3	0.7704	
	GFP++	3	0.0087	**
	GFP+++	3	3.2429E-05	****

Figure 3G				
		N value	exact *p* value	asterics
2 day	UNT	7	N/A	
	GFP-+	7	0.2742	ns
	GFP++	7	0.00006	****
	GFP+++	4	3.74249E-12	****
15 days	UNT	4	N/A	
	GFP-+	4	0.9277	ns
	GFP++	4	0.0907	ns
	GFP+++	4	0.5048	ns

Figure 4B				
		N value	exact *p* value	asterics
Basal	UNT	5	N/A	
	GFP-+	5	0.0021	**
	GFP++	5	0.0268	*
	WT	4	0.0005	***
Maximal	UNT	6	N/A	
	GFP-+	5	0.0011	**
	GFP++	5	0.0321	*
	WT	4	0.0011	**
Spare capacity	UNT	6	N/A	
	GFP-+	5	0.0012	**
	GFP++	5	0.0494	*
	WT	4	0.0055	**
ATP-linked	UNT	6	N/A	
	GFP-+	5	0.0006	***
	GFP++	5	0.0178	*
	WT	4	0.0002	***

Figure 4C				
		N value	exact *p* value	asterics
Coupling efficiency (%)	UNT	6	N/A	
	GFP-+	5	0.0003	***
	GFP++	5	0.0002	***
	WT	4	0.0002	***

Figure 4D				
		N value	exact *p* value	asterics
Mitochondrial Protein synthesis	UNT	4	N/A	
	GFP-+	5	0.0589	ns
	GFP++	5	0.0026	**
	WT	2	N/A	

Figure 4E				
		N value	exact *p* value	asterics
NDUFB8	UNT	6	N/A	
	GFP-+	6	0.075	ns
	GFP++	6	0.0315	*
COXI	UNT	6	N/A	ns
	GFP-+	6	0.0211	*
	GFP++	6	0.1533	ns
SDHA	UNT	6	N/A	ns
	GFP-+	6	0.4805	ns
	GFP++	6	0.5787	ns

Fig. EV1D				
		N value	exact *p* value	asterics
1 day	UNT	10	N/A	
	GFP-+	9	0.5635	ns
	GFP++	9	0.0023	**
15 days	UNT	4	N/A	
	GFP-+	4	0.7572	ns
	GFP++	3	9.00458E-05	****
