# Supplementary material for: mitoTev‐TALE: a monomeric DNA editing enzyme to reduce mutant mitochondrial DNA levels
Source: EMBO Mol Med. 2018 Jul 16;10(9):e8084. doi: 10.15252/emmm.201708084 (PMC6127889; doi:10.15252/emmm.201708084)
Supplement: Supplementary file 2 — Expanded View Figures PDF [file EMMM-10-e8084-s002.pdf]

Expanded View Figures

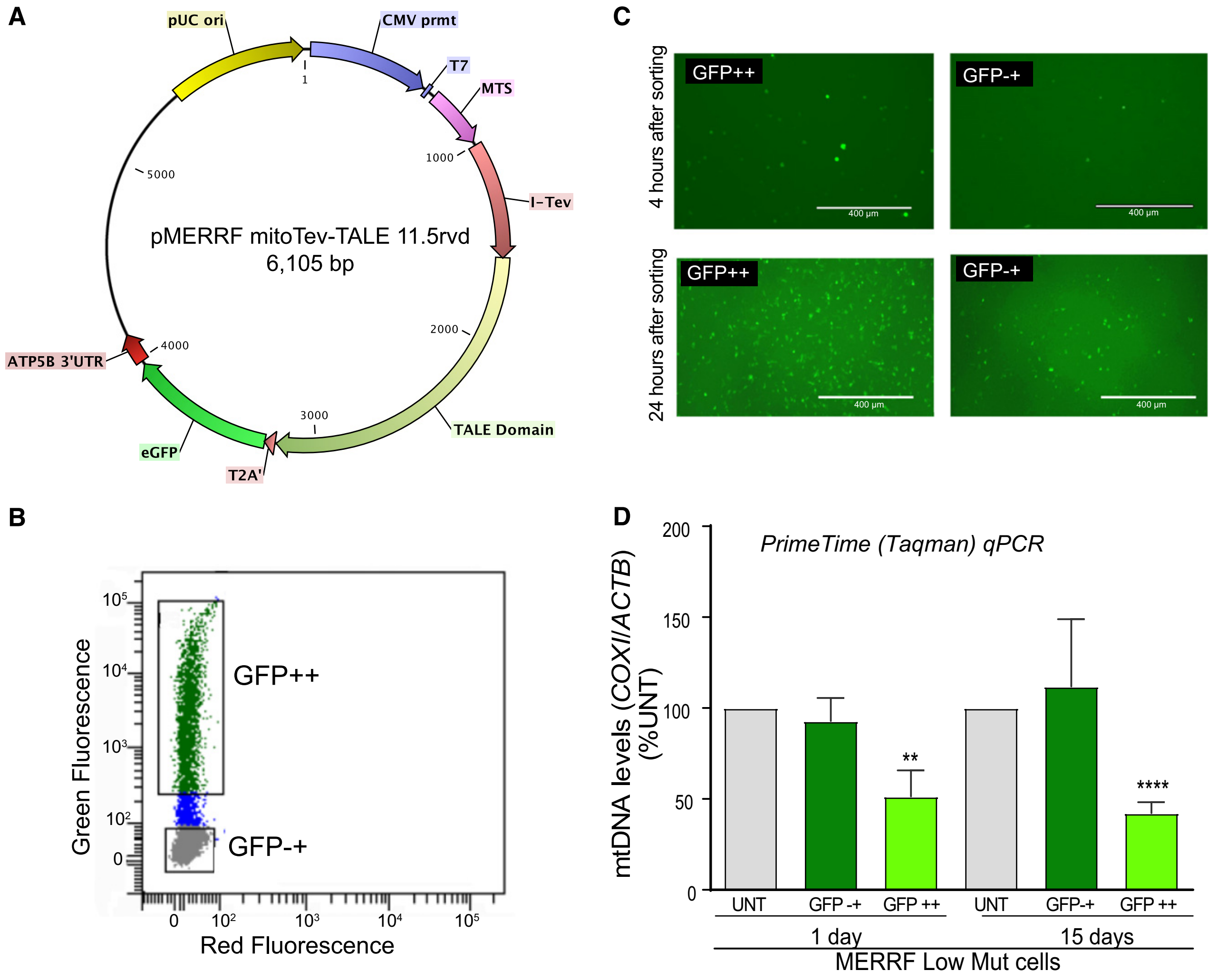

Figure EV1. Cell sorting of cybrids transfected with the plasmid coding for the MERRF mitoTev-TALE.

A Plasmid expressing the MERRF mitoTev-TALE, showing the location of the eGFP gene.

B FACS gating of cells transfected (24 h) and sorted by GFP fluorescence. Cells sorted as positive for the eGFP marker were termed "GFP++", and cells without detectable fluorescence were termed "GFP−+".

C Fluorescence microscopy showing the presence of some GFP-positive cells in the GFP−+ population (4 and 24 h after sorting).

D Determination of the total mtDNA levels 1 and 15 days after transfection in clone 7 from the first set of sortings. The graph shows the ratio of (COXI/ACTB) PrimeTime probes. Data are mean ± SEM of n = 9–10, 1 day; n = 3–4, 15 days. Statistical analysis was performed using two-tailed Student's t-test between two groups (UNT. vs GFP−+ or UNT. vs GFP++, $^{**}P < 0.005$; $^{****}P < 0.0001$).

© 2018 The Authors

MitoTev-TALE (8.5 RVD)

MSVLTPLLLRGLTGSARRLPVPRAKIHSLPPEGKLMASTRVLASRLASQMAASAKVARPAVRVA
QVSKRTIQTGSPLQTLKRTQMTSIVNATTRQAFQKRAYSSPAGMGKSGIYQIKNTLNNKVYVGS
AKDFEKRWKRHFKDLEKGCHSSIKLQRSFNKHGNVFECSILEEIPYEKDLIIERENFWIKELNSKI
NGYNIADATFGDTCSTHPLKEEIIKKRSETVKAKMLKLGPDGRKALYSKPGSGDIADLRTLGYSQ
QQQEKIKPKVRSTVAQHHEALVGHGFTHAHIVALSQHPAALGTVAVKYQDMIAALPEATHEAIV
GVGKQWSGARALEALLTVAGELRGPPLQLDTGQLLKIAKRGGVTAVEAVHAWRNALTGAPLNL
TPQQVVAIASNNGGKQALETVQALLPVLCQAHGLTPQQVVAIASNNGGKQALETVQRLLPVLC
QAHGLTPEQVVAIASHDGGKQALETVQRLLPVLCQAHGLTPQQVVAIASNGGGKQALETVQRL
LPVLCQAHGLTPEQVVAIASHDGGKQALETVQRLLPVLCQAHGLTPQQVVAIASNGGGKQALE
TVQRLLPVLCQAHGLTPEQVVAIASHDGGKQALETVQALLPVLCQAHGLTPQQVVAIASNGGG
KQALETVQRLLPVLCQAHGLTPQQVVAIASNGGGRPALESIVAQLSRPDPALAALTNDHLVALA
CLGGRPALDAVKKGLG

MitoTev-TALE (11.5 RVD)

MSVLTPLLLRGLTGSARRLPVPRAKIHSLPPEGKLMASTRVLASRLASQMAASAKVARPAVRVA
QVSKRTIQTGSPLQTLKRTQMTSIVNATTRQAFQKRAYSSPAGMGKSGIYQIKNTLNNKVYVGS
AKDFEKRWKRHFKDLEKGCHSSIKLQRSFNKHGNVFECSILEEIPYEKDLIIERENFWIKELNSKI
NGYNIADATFGDTCSTHPLKEEIIKKRSETVKAKMLKLGPDGRKALYSKPGSGDIADLRTLGYSQ
QQQEKIKPKVRSTVAQHHEALVGHGFTHAHIVALSQHPAALGTVAVKYQDMIAALPEATHEAIV
GVGKQWSGARALEALLTVAGELRGPPLQLDTGQLLKIAKRGGVTAVEAVHAWRNALTGAPLNL
TPQQVVAIASNNGGKQALETVQALLPVLCQAHGLTPQQVVAIASNNGGKQALETVQRLLPVLC
QAHGLTPEQVVAIASHDGGKQALETVQRLLPVLCQAHGLTPQQVVAIASNGGGKQALETVQRL
LPVLCQAHGLTPEQVVAIASHDGGKQALETVQRLLPVLCQAHGLTPQQVVAIASNGGGKQALE
TVQRLLPVLCQAHGLTPEQVVAIASHDGGKQALETVQALLPVLCQAHGLTPQQVVAIASNGGG
KQALETVQRLLPVLCQAHGLTPQQVVAIASNGGGKQALETVQRLLPVLCQAHGLTPEQVVAIAS
NIGGKQALETVQRLLPVLCQAHGLTPEQVVAIASNIGGKQALETVQRLLPVLCQAHGLTPQQVV
AIASNGGGRPALESIVAQLSRPDPALAALTNDHLVALACLGGRPALDAVKKGLGDL

Figure EV2. Protein sequence of mitoTev-TALEs.

The I-TevI domain composed of 137 amino acids is highlighted in blue; the TALE N-terminal is highlighted in green, and the respective RVDs (either 8.5 or 11.5) are highlighted in yellow.

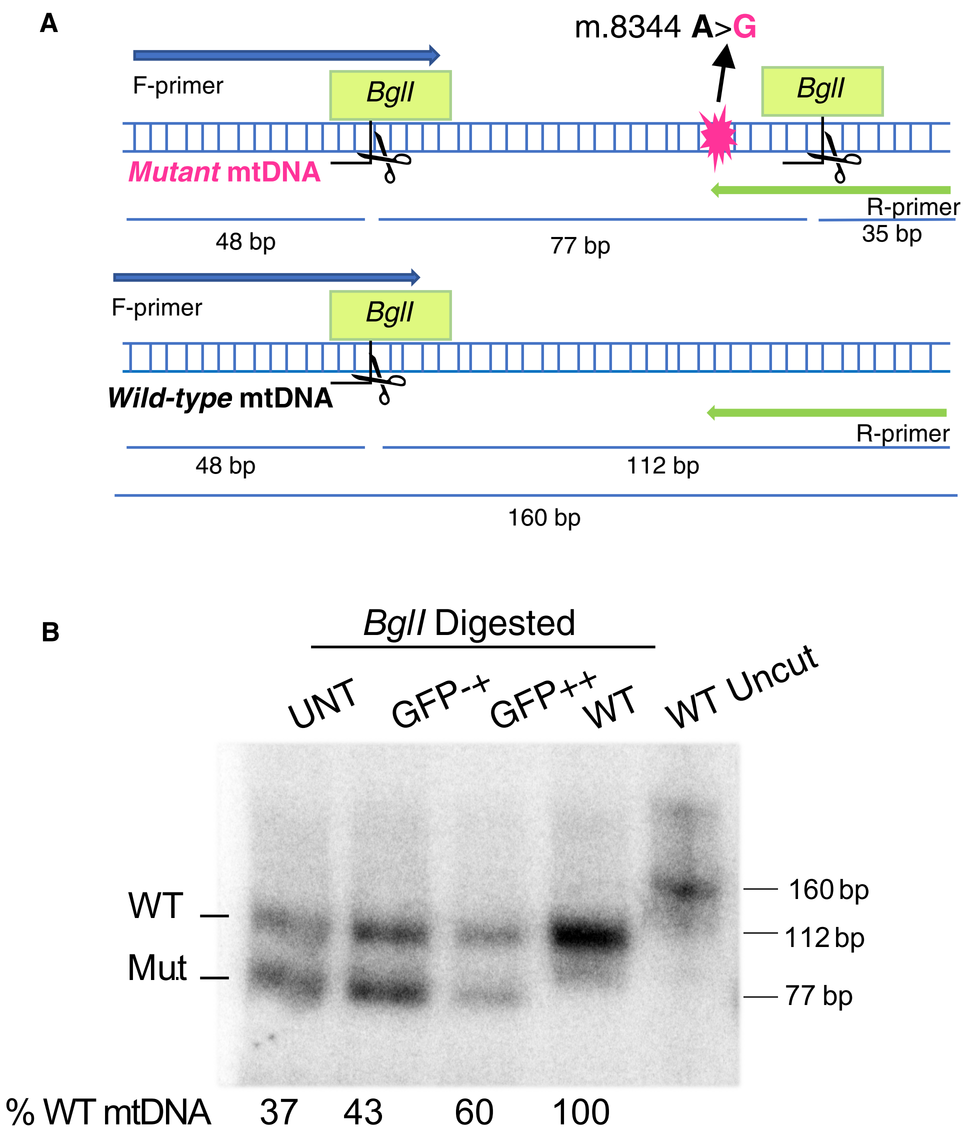

Figure EV3. Alternative RFLP for the detection of the m.8344A>G mtDNA mutation.

A Schematic representation of the mtDNA region surrounding the m.8344A>G. The R-primer (green) creates a *Bgl*I site only in the mutant mtDNA sequence, which allows the determination of the mtDNA heteroplasmy in the tested cells. The F-primer adds an additional *Bgl*I site, assuring that digestion is complete.

B After *Bgl*I digestion, the "last-cycle hot" labeled DNA samples are electrophoresed in a 12% polyacrylamide gel and exposed to a phosphorimager screen.

© 2018 The Authors
