# Supplementary figures and images for: mitoTev‐TALE: a monomeric DNA editing enzyme to reduce mutant mitochondrial DNA levels
Source: EMBO Mol Med. 2018 Jul 16;10(9):e8084. doi: 10.15252/emmm.201708084 (PMC6127889; doi:10.15252/emmm.201708084)

C

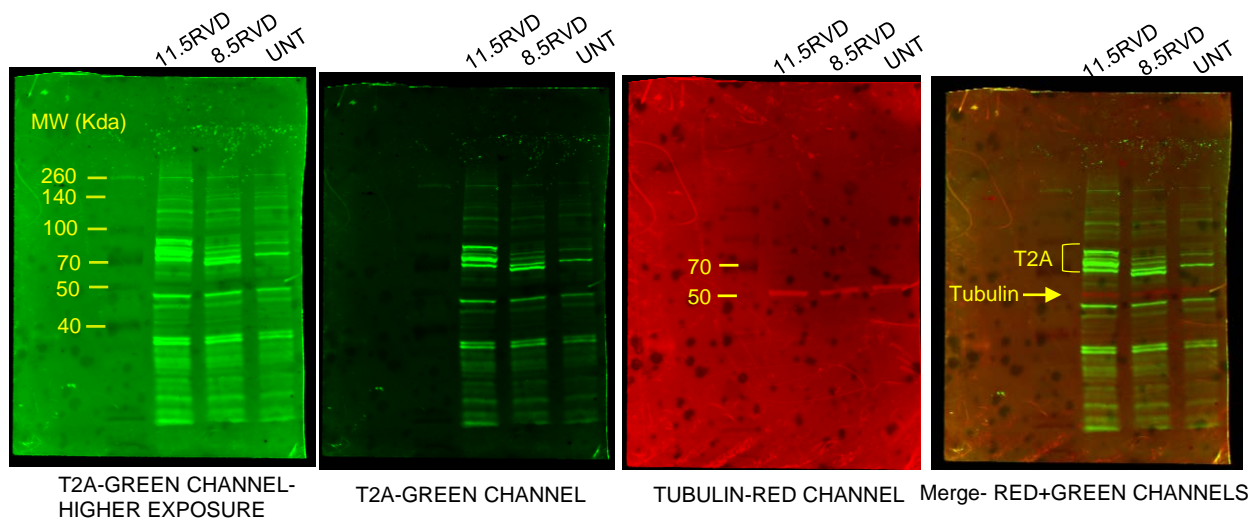

D

11.5RVD MERRF mitoTev-TALE

8.5RVD MERRF mitoTev-TALE

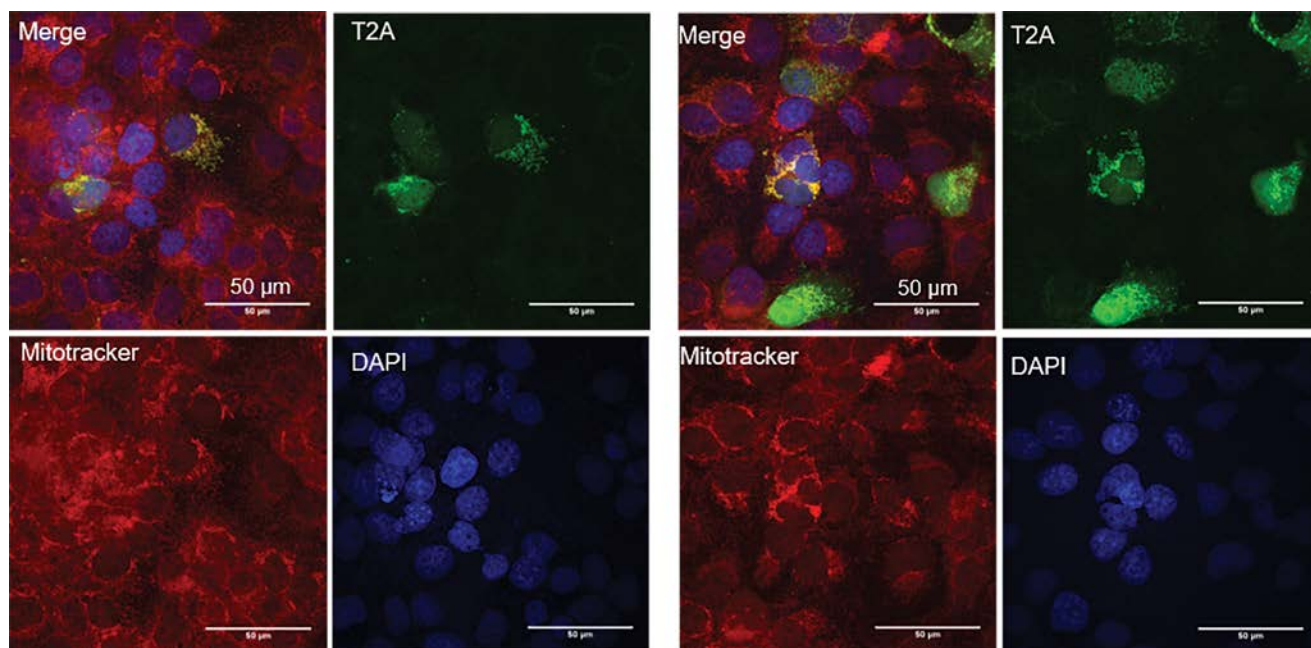

Supplement: Supplementary file 4 — Source Data for Figure 1 [file EMMM-10-e8084-s003.pdf]

A

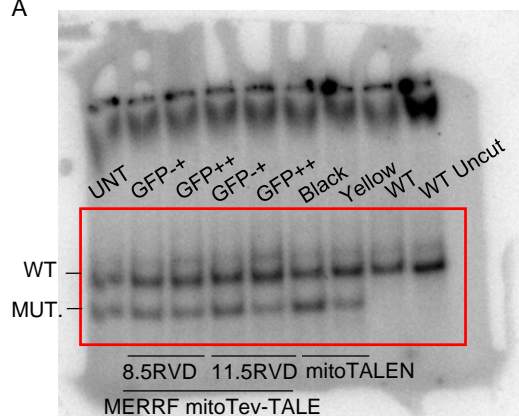

C

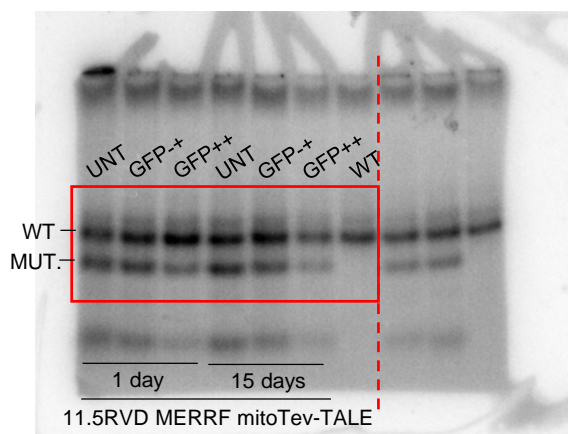

Supplement: Supplementary file 5 — Source Data for Figure 2 [file EMMM-10-e8084-s004.pdf]

A

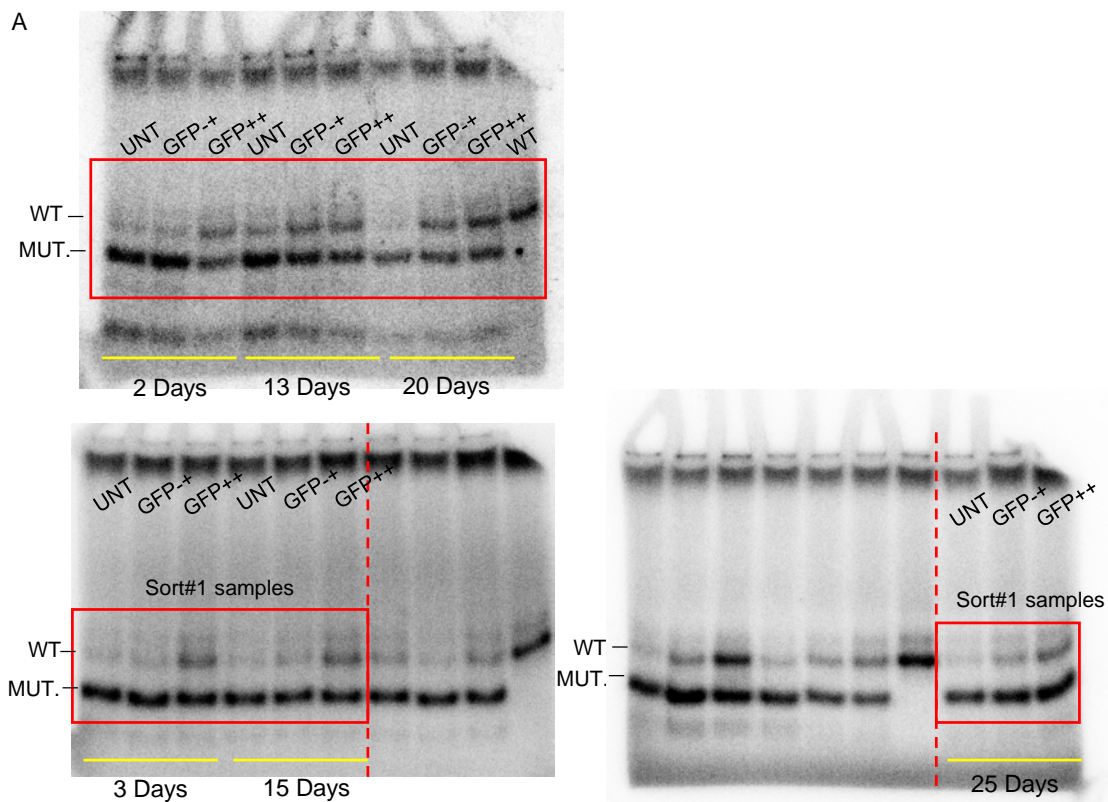

Supplement: Supplementary file 6 — Source Data for Figure 3 [file EMMM-10-e8084-s005.pdf]

D

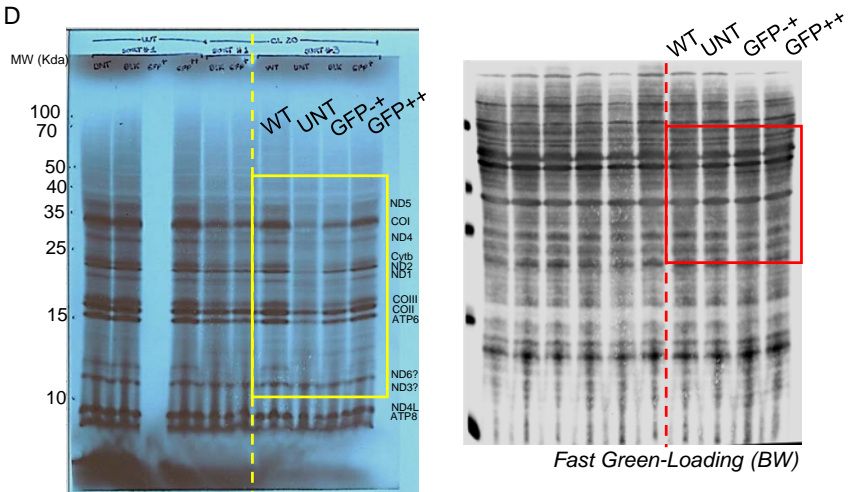

E

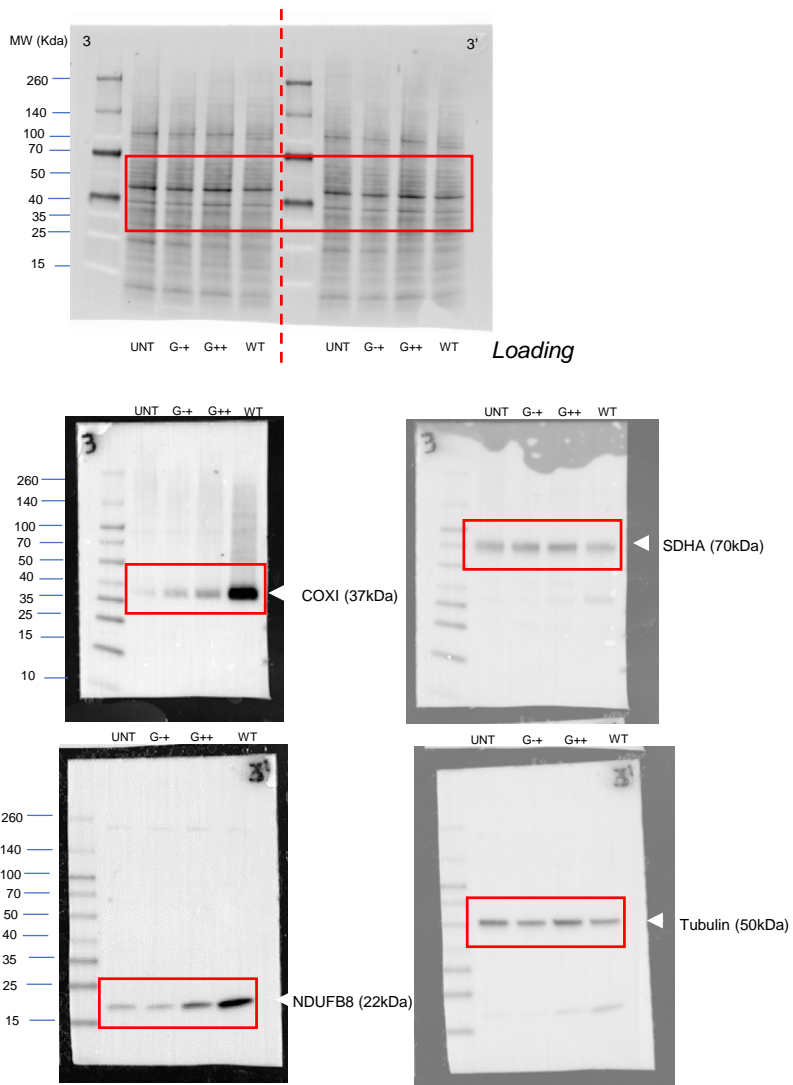

Supplement: Supplementary file 7 — Source Data for Figure 4 [file EMMM-10-e8084-s006.pdf]
